# Light-Induced Triplet–Triplet Electron Resonance Spectroscopy

**DOI:** 10.1021/acs.jpclett.0c02884

**Published:** 2020-12-11

**Authors:** Arnau Bertran, Kevin B. Henbest, Marta De Zotti, Marina Gobbo, Christiane R. Timmel, Marilena Di Valentin, Alice M. Bowen

**Affiliations:** † Centre for Advanced Electron Spin Resonance and Inorganic Chemistry Laboratory, Department of Chemistry, University of Oxford, South Parks Road, Oxford OX1 3QR, United Kingdom; ‡ Department of Chemical Sciences, University of Padova, Via Marzolo 1, 35131 Padova, Italy; § Department of Chemistry and Photon Science Institute, University of Manchester, Oxford Road, Manchester M13 9PL, United Kingdom

## Abstract

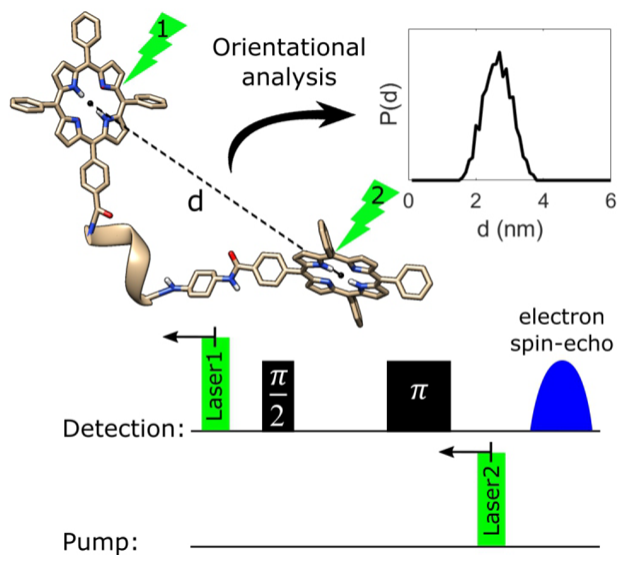

We present a new technique, light-induced triplet–triplet electron resonance spectroscopy (LITTER), which measures the dipolar interaction between two photoexcited triplet states, enabling both the distance and angular distributions between the two triplet moieties to be determined on a nanometer scale. This is demonstrated for a model bis-porphyrin peptide that renders dipolar traces with strong orientation selection effects. Using simulations and density functional theory calculations, we extract distance distributions and relative orientations of the porphyrin moieties, allowing the dominant conformation of the peptide in a frozen solution to be identified. LITTER removes the requirement of current light-induced electron spin resonance pulse dipolar spectroscopy techniques to have a permanent paramagnetic moiety, becoming more suitable for in-cell applications and facilitating access to distance determination in unmodified macromolecular systems containing photoexcitable moieties. LITTER also has the potential to enable direct comparison with Förster resonance energy transfer and combination with microscopy inside cells.

Determining the structure and dynamics of complex biological macromolecular systems is one of the current challenges in the physical and life sciences. Electron spin resonance (ESR) pulse dipolar spectroscopy (PDS) has become an important tool for this purpose.^1^ By measuring the electron–electron dipolar interaction between two paramagnetic species, ESR PDS techniques allow for the determination of the distance distributions and in some cases the relative orientations of the paramagnetic centers, giving direct insight into conformational dynamics.^2^ The biologically relevant distance range of 1.5–8 nm can be accessed with high precision and reliability, with an upper limit that can be extended to 16 nm for two stable paramagnetic centers by full deuteration of both the sample and the solvent.^3,4^

The most commonly used PDS technique is double electron–electron resonance (DEER) on nitroxide spin-labels, which have been attached to the molecular system of interest by site-directed spin labeling or chemical modification.^5−7^ A modified version of this experiment, four-pulse light-induced double electron–electron resonance (LiDEER), was developed using the photoexcited triplet state (*S* = 1) of a 5-(4′-carboxyphenyl)-10,15,20-triphenylporphyrin moiety (TPP), formed by a laser flash before the microwave (MW) pulse sequence, as the detection spin and a nitroxide radical as the pump spin.^8,9^ The spin polarization of the porphyrin triplet state,^10,11^ due to a non-Boltzmann population of the triplet state sublevels resulting from the intersystem crossing (ISC) process,^12^ provided enhanced sensitivity. This technique was successfully applied to a synthetic model peptide ruler with porphyrin–nitroxide distances ranging from 1.8 to 8.1 nm, rendering spin–spin distance distributions in good agreement with the values predicted by density functional theory (DFT) calculations.^9^ LiDEER was later extended to protein systems containing endogenous protoporphyrin groups and a light-harvesting pigment cluster, orthogonally spin-labeled with nitroxides,^13,14^ and also proteins binding exogenous porphyrins.^15^ Other triplet states, including fullerenes, have also been used as spin-labels.^16^

Alternatively, it has been shown that the dipolar interaction between a triplet state and a nitroxide spin can also be detected using a time-varying laser flash moving through a Hahn echo MW sequence, which acted as pump to form the porphyrin triplet, in a technique named laser-induced magnetic dipole spectroscopy (LaserIMD).^17,18^ Pumping on the triplet in this fashion has the potential to lead to enhanced modulation depths compared to those achieved with LiDEER, as it is effectively possible to excite the complete triplet spectrum, therefore removing the limitation of the achievable bandwidth of the MW pulses.^17^ LaserIMD benefits from being a single-frequency experiment, which enables a higher cavity *Q*-factor to be used to enhance experimental sensitivity by reducing pulse length. While a primary echo acquisition can be used, for short interspin distances the intrinsic uncertainty in the determination of the zero time of the LaserIMD experiment can lead to artifacts in the distance distribution.^13^ A refocused version of LaserIMD (ReLaserIMD) has been used to address this problem by providing a symmetric dipolar trace with respect to the zero time, which is analogous to four-pulse DEER. LaserIMD was applied to both model peptides and protoporphyrin proteins labeled with nitroxides.^13,17,19^

It has been shown that the performances of LiDEER and LaserIMD are complementary to one another; depending on the system studied, one may be preferential.^18,19^ Additionally, a light-induced modification of the relaxation-induced dipolar modulation enhancement (RIDME) experiment, known as LiRIDME, has also been demonstrated to work for systems in which there are differences in longitudinal relaxation time between the radical and triplet.^13^

However, the aforementioned techniques are limited to the measurement of the dipolar interaction between a permanent paramagnetic center and a photoexcited triplet and cannot access dipolar interactions between two photoexcitable centers. Detection of dipolar interactions between two light-induced paramagnetic centers would afford the possibility of measuring distances in macromolecules, containing suitable chromophores, which are ESR silent, and thus undetectable, in their ground states.

Here we present a new light-induced ESR PDS technique, light-induced triplet–triplet electron resonance spectroscopy (LITTER), which enables the measurement of the dipolar interaction between two photoexcited triplet states and the determination of the distance distribution between the two triplet-bearing moieties. This technique removes the restriction of having to use a permanent paramagnetic center in LiDEER, (Re)LaserIMD, and LiRIDME. LITTER combines the advantages of both LiDEER and LaserIMD: the detection of a spin-polarized triplet state formed by an initial laser flash, the use of the short Hahn echo detection sequence, and the unlimited pump bandwidth afforded by using a second variable time laser flash to form the second triplet (Figure 1). The second laser flash switches on the dipolar interaction and gives rise to a time-dependent modulation of the primary echo intensity (Figure 1b).

**Figure 1:**
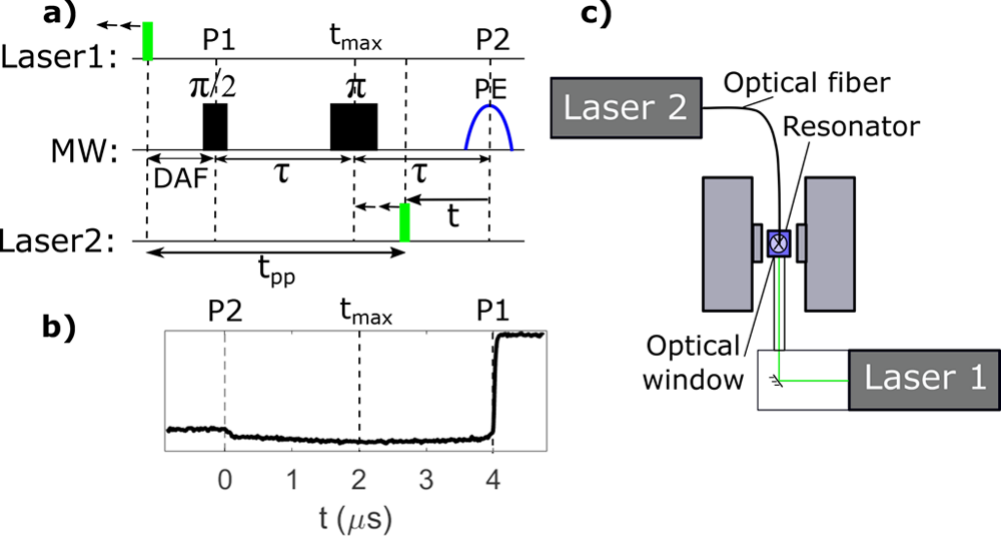
LITTER experiment. (a) Pulse sequence and (b) spin–echo intensity time trace (in arbitrary units) for the bis-porphyrin model system TPP-Ala-[(αMe)Val]_4_-NH-C_6_H_10_-NH-TPP (**1**) (see Figure 2 for the molecular structure). At 0 μs (P2), laser 2 crosses the primary echo (PE). When *t* > 0, a drop in the echo intensity occurs due to the change in the local magnetic field on the detection triplet caused by the formation of the second triplet during the free evolution time. At *t* = 2 μs (*t*_max_, maximum free evolution time), laser 2 crosses the π pulse, which corresponds to the minimum in the time trace. Finally, at *t* = 4 μs (P1), laser 2 crosses the π/2 pulse, so when *t* > 4 μs, the triplet spins formed by both lasers are fully refocused at the PE, leading to an increase in signal intensity and the disappearance of modulation. The time delay between the two laser flashes (*t*_pp_) is kept constant throughout the experiment, while the delay after flash (DAF) is increased stepwise. (c) Schematic diagram of the experimental setup. The experiment is achieved by synchronizing two laser sources with the pulse ESR spectrometer via delay pulse generators (see the Supporting Information for experimental details and optimization of experimental conditions).

In this Letter, we report the results of the novel LITTER experiment on the bis-porphyrin model peptide TPP-Ala-[(αMe)Val]_4_-NH-C_6_H_10_-NH-TPP **[1]** (Figure 2, red). The photoexcitation of both porphyrin moieties in the molecule was performed at 512 nm, corresponding to the most intense absorption maximum of the TPP Q-band (Figure S1). The LITTER trace recorded for bis-porphyrin **[1]** was compared to a LITTER trace recorded using the same experimental settings but with the single-porphyrin peptide TPP-(Ala-Aib)_6_-Ala-OH **[2]** as a control (Figure 2, black). The trace of system **[2]** did not show a modulation and displayed only a slow decay. This background decay results from the combination of longitudinal relaxation from the nascent non-Boltzmann population, decay of the triplet state, and intermolecular dipolar interactions. The absence of modulation in **[2]** proves that the effect observed in **[1]** is due to the intramolecular dipolar interaction between the two photoexcited triplet states.

**Figure 2:**
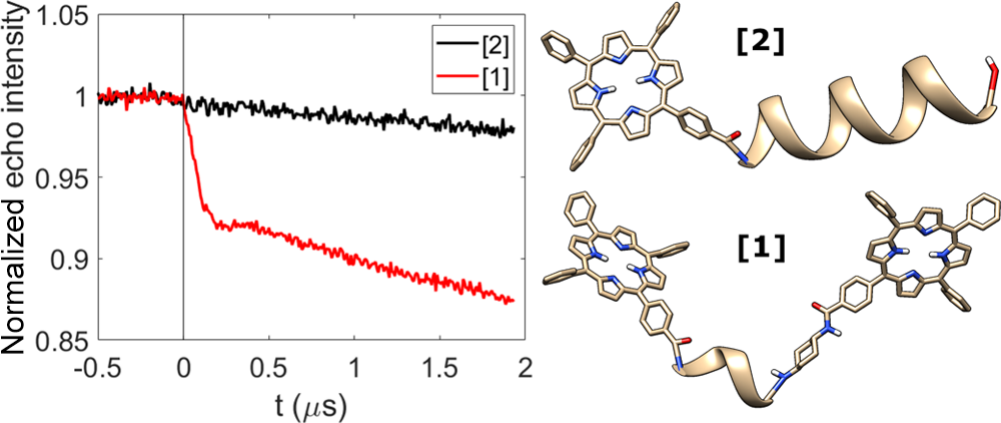
LITTER traces (left) for the bis-porphyrin peptide model system TPP-Ala-[(αMe)Val]_4_-NH-C_6_H_10_-NH-TPP **[1]** (red) and the single-porphyrin peptide TPP-(Ala-Aib)_6_-Ala-OH **[2]** used as control (black). Amino acid key: Ala (l-alanine), (αMe)Val (l-α-methyl valine), and Aib (α-aminoisobutyric acid). The external magnetic field was set to the emissive maximum of the photoexcited triplet spectrum, and only the first half of the LITTER trace was recorded, using a τ of 2004 ns and a *t*_pp_ of 6.82 μs. Lowest-energy geometries (right) of molecules **[1]** and **[2]** optimized by DFT, with an expected distance between the centers of the two porphyrins in **[1]** of 2.6 nm.

The zero-field splitting (ZFS) tensor for a TPP moiety is significantly anisotropic. LITTER traces (Figure 3b) acquired on the turning points of the photoexcited triplet state spectrum corresponding to the Y^–^ and Z^–^ canonical orientations of the ZFS (Figure 3a) demonstrate significant orientation selection. Orientation selection is well reported for ESR PDS experiments measured between two stable spin centers where at least one has an anisotropic **g** tensor. These can be modeled using orientation-dependent simulations.^20−22^ Orientation selection arises from the limited bandwidth of the microwave pulses used for detection relative to the complete spectral width of the porphyrin triplet, such that only a small number of molecular orientations are excited. Previous examples of orientation selection in light-induced ESR include the application to hyperfine spectroscopy, which was used to determine the relative orientation of the ZFS tensor to the hyperfine tensors within the molecular structure.^23−25^

**Figure 3:**
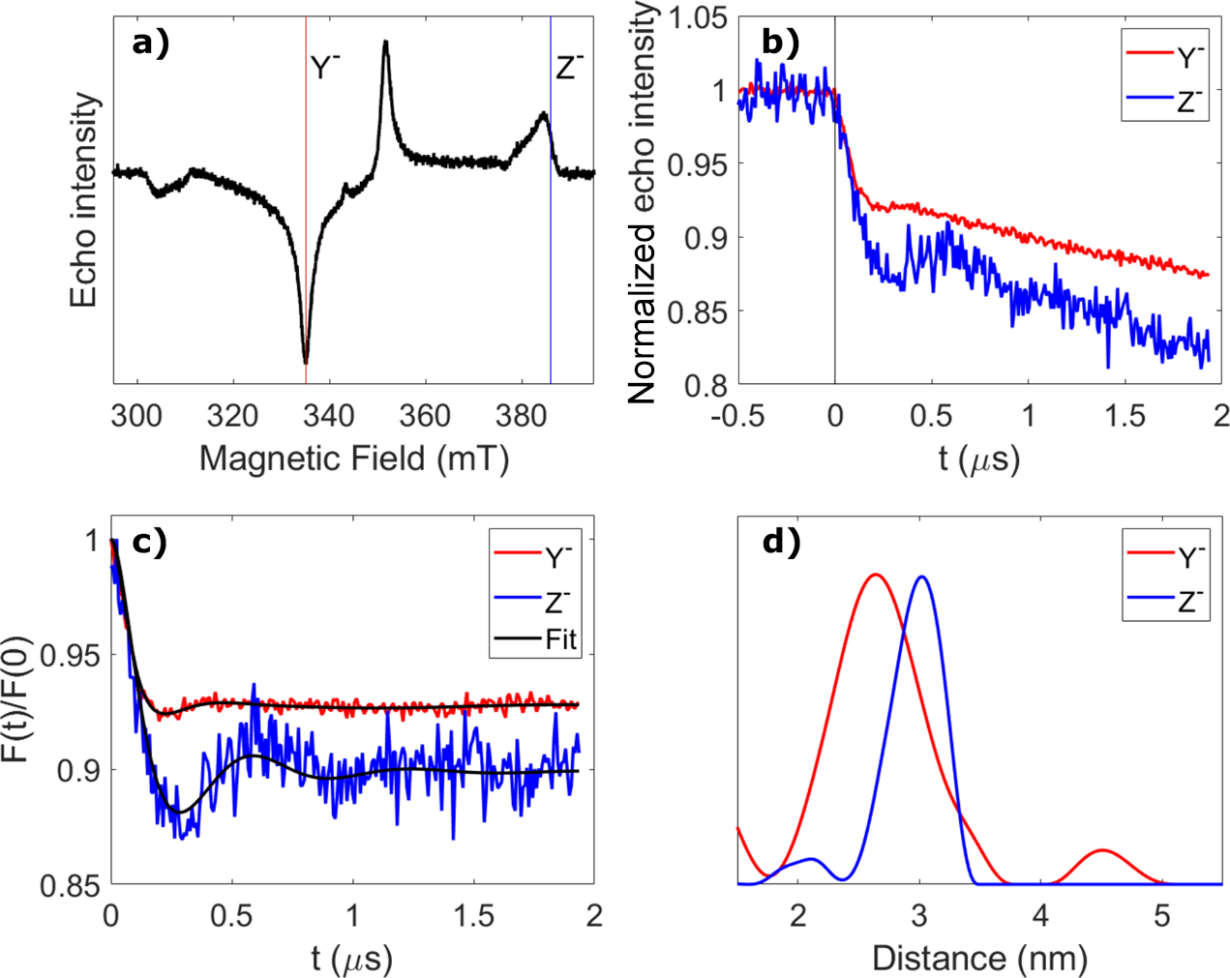
LITTER results for molecule **[1]** measured on Y^–^ and Z^–^. (a) Field-swept electron spin–echo spectrum after laser flash, showing the Y^–^ (red) and Z^–^ (blue) turning points. (b) Raw LITTER traces. (c) Form factors with the corresponding fits obtained with *DeerAnalysis*, showing modulation depths of 8% and 13%. (d) Resulting spin–spin distance distributions, with maxima at 2.65 nm (red) and 3.02 nm (blue).

Quantitative analysis of ESR PDS is frequently obtained using *DeerAnalysis* to determine distance distributions from the experimentally recorded time traces.^26^ This program uses Tikhonov regularization with an orientation-independent dipolar interaction model. For the Z^–^ and Y^–^ LITTER traces, this analysis yielded apparent distance distributions (Figure 3d). These apparent distance distributions show some agreement with the optimized structures of **[1]** generated by DFT, where two local minima were identified, named **[1]***bent* and **[1]***extended* conformers, with center-to-center interporphyrin distances of 2.6 and 3.4 nm, respectively (Figure S7). To determine if either of these models is consistent with both Z^–^ and Y^–^ LITTER data sets, it was necessary to perform orientation-dependent simulations considering the ZFS anisotropy in combination with orientation selection of the detection MW pulses.

Orientation-dependent LITTER simulations were carried out using a modified form of asimulation algorithm described previously (see the Supporting Information for further details).^27^ The simulation with the model **[1]***bent* conformation rendered good fits to both Z^–^ and Y^–^ LITTER data sets (Figure 4b), with the positions of the pump spin centers contributing to the simulated form factors reasonably distributed around the geometry of minimum energy (Figure 4a). The relative orientation of the pump spin center with respect to the spin–spin vector was found to have a negligible effect on the results of the simulation, as expected from the effectively infinite spin excitation bandwidth of the laser flash. The resulting spin–spin distance distribution was in good agreement with that obtained from the orientationally independent analysis of the Y^–^ data set (Figure 4c), showing that the longer spin–spin distances obtained from Z^–^ were indeed an orientation artifact. The results of the analogous simulation with the model **[1]***extended* conformation did not fit the experimental LITTER data sets (Figure S10b), which indicated that the model **[1]***extended* conformer was not the dominant conformer in a frozen *d*_6_-ethanol solution. This conclusion was reaffirmed by the results of a third simulation considering both **[1]***bent* and **[1]***extended* models simultaneously, which rendered positions of the pump spin center clustered around the **[1]***bent* conformation (Figure S11). The prevalence of the more stable **[1]***bent* conformer in a frozen solution is consistent with the energetics of *bent–extended* interconversion obtained by DFT [Figure S8, dihedral A (blue)], with an energy barrier (10.7 kJ/mol) much larger than the thermal energy at room temperature (0.068 kJ/mol). Major rotations of the porphyrin moieties with respect to the rest of the molecule are expected to be energetically unfavorable [Figure S8, dihedrals B (green) and C (red)], suggesting that the distribution of pump spin center positions may originate mainly from the flexibility of the peptide spacer.

**Figure 4:**
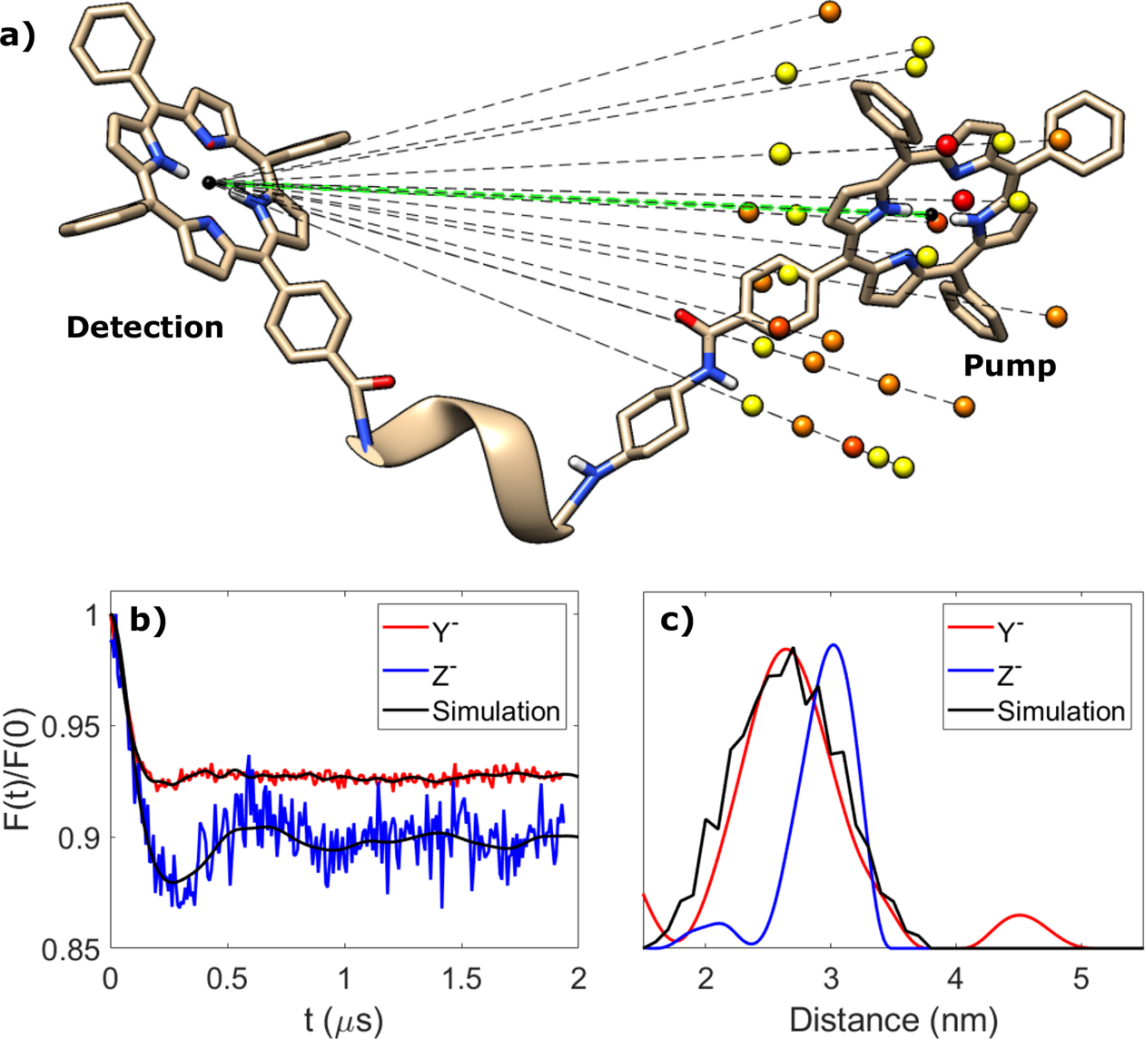
Orientation-dependent LITTER simulation for the **[1]***bent* conformer. (a) DFT-optimized geometry with the different positions of the pump spin center contributing to the simulated form factor shown as spheres representing the centers of the corresponding porphyrins. The relative weight of each pump position in the simulated form factor is represented by the color of the sphere (yellow, 1; orange, 2; dark orange, 3; red, 4). The dipolar vector between the centers of the two porphyrins of the DFT-optimized geometry is highlighted in green. Hydrogen atoms have been omitted for the sake of clarity. (b) Form factors obtained experimentally (red and blue) and from orientation-dependent simulations (black). (c) Spin–spin distance distributions obtained using *DeerAnalysis* (red and blue) and from orientation-dependent simulations (black).

The version of LITTER presented in this Letter uses only a single color of light excitation. While this has the potential to be advantageous if only a single laser source with a suitable delay line is available, it also imposes a limitation on the observable modulation depth. Assuming complete excitation of all ground state porphyrin (TPP) moieties in the sample by both laser flashes, a maximum modulation depth of 16% would be observed with a triplet state quantum yield of 0.8 (see the Supporting Information for further details). In addition, the similar extinction coefficients of ^1^TPP and ^3^TPP at the excitation wavelength used in this study may allow additional photoexcitation of ^3^TPP by the second laser flash,^28^ leading to a partial loss of detection triplet spins.^29^ These limitations could be overcome by a two-color version of LITTER, replacing one of the porphyrins by a suitable optically orthogonal photoexcitable moiety.^30^

Conversely, theability to measure the dipolar interaction and thereby structural information between two chromophores of the same type is useful for systems in which orthogonal labeling is difficult, for example, homodimeric systems. In such systems, Förster resonance energy transfer (FRET) between two chromophores of the same type is possible only by observing fluorescence depolarization.^31^ However, this effect is observed only when the chromophores adopt different orientations relative to the polarization of the light. Such a limitation does not apply to LITTER, and furthermore, LITTER can be used to directly measure the relative orientation of the chromophores, information that cannot be obtained from a FRET experiment.

In conclusion, we have demonstrated that LITTER allows the measurement of the dipolar interaction between the photoexcited triplet states of two free-base porphyrin moieties separated by a short peptide spacer. The dipolar traces obtained show strong orientation effects due to the large anisotropy in the ZFS of the photoexcited porphyrin triplet state, rendering different apparent distance distributions upon analysis with software that uses an orientation-independent kernel.^26^ Using orientation-dependent simulations complemented with DFT calculations, we have successfully described these orientation effects and obtained the real spin–spin distance distribution and information about the dominant conformation of the bis-porphyrin molecule in a frozen solution. The large anisotropy of the triplet state ZFS and the resulting strong orientation dependence of the LITTER experiment have the potential to distinguish between small differences in orientation of two chromophores in rigid systems. Although orientation dependence can also be observed in ESR PDS of metal-containing systems,^20,32−35^ photoexcited triplet states have proven to be superior for detection due to their strong spin polarization and more favorable relaxation properties.^9^ Additionally, the single-frequency nature of LITTER removes the restriction of resonator bandwidth, which often prevents the complete exploitation of the orientation dependence in bis-stable radical systems. Finally, the origin of the orientation dependence is the ZFS, removing the need to use high field, which is often required for greater orientation resolution in spin-half paramagnetic centers.

Our LITTER technique removes the restriction, recurrent in all of the light-induced ESR PDS techniques reported so far, of having to use one stable paramagnetic center to perform distance measurements. By accessing the dipolar interaction between two photoexcited triplet states, LITTER has the potential to enable distance distributions and relative orientations to be obtained for unmodified macromolecular systems containing endogenous photoexcitable moieties, such as light-harvesting proteins, heme proteins and flavoproteins, or systems modified with several photoexcitable triplet spin-labels. This technique could also be used in combination with FRET spectroscopy if suitable photoexcitable moieties are chosen, exploiting the complementary features of the two techniques.

In a manner similar to that for FRET, which has found significant applications in cells,^36^ we predict that the novel LITTER technique is a strong candidate for measuring structural information between chromophores in the cellular environment, using chromophore triplet states that can be generated within cells.^37^ Removing the need to have a permanent paramagnetic center, such as nitroxide spin-labels, which are known to be unstable and rapidly degraded inside cells,^38,39^ is a key point for the success of in-cell structural studies and also mitigates the need to use more complex spin-labels such as trityl radicals or gadolinium complexes.^40−42^ Furthermore, the combination of LITTER with microscopy techniques might facilitate the observation of structural changes for a system in different parts of a cell, for instance, by using microscopy to pinpoint the location of the chromophores within the cell before using LITTER to probe structural parameters. Consequently, LITTER is an exciting new technique with the potential to cause a step change in the application of ESR PDS to biological systems.

## Methods

The experimental and computational methods are described in the Supporting Information.

## Supporting Information Available

Experimental methods, including synthesis, sample preparation, and ESR spectroscopy; computational methods, including density functional theory calculations and orientation-dependent simulations; and results, including spectroscopic characterization of peptide **[1]**, optimization of experimental conditions for LITTER, computational results, and modulation depth analysis (PDF)
