# Supplementary material for: Light-Induced Triplet–Triplet Electron Resonance Spectroscopy
Source: J Phys Chem Lett. 2020 Dec 11;12(1):80–5. doi: 10.1021/acs.jpclett.0c02884 (PMC8016185; doi:10.1021/acs.jpclett.0c02884)
Supplement: Supplementary file 1 — jz0c02884_si_001.pdf [file jz0c02884_si_001.pdf]

Supporting Information for

Light-Induced Triplet-Triplet Electron Resonance Spectroscopy

Arnau Bertran,[1] Kevin B. Henbest,[1] Marta De Zotti,[2] Marina Gobbo,[2] Christiane R. Timmel,[1] Marilena Di Valentin,[,2] and Alice M. Bowen*[,3,1]*

[1]Center for Advanced Electron Spin Resonance and Inorganic Chemistry Laboratory, Department of Chemistry, University of Oxford, South Parks Road, Oxford OX1 3QR, United Kingdom.

[2]Department of Chemical Sciences, University of Padova, Via Marzolo 1, 35131 Padova, Italy.

[3] Department of Chemistry and Photon Science Institute, University of Manchester, Oxford Road, Manchester M13 9PL, United Kingdom.

CONTENTS

S1. Experimental methods

S1.1. Synthesis

S1.2. Sample preparation

S1.3. ESR spectroscopy

S2. Computational methods

S2.1. Quantum mechanical calculations

S2.2. Orientation dependent simulations

S3. Results

S3.1. Spectroscopic characterization of peptide [1]

S3.2. Optimization of experimental conditions for LITTER

S3.3. Computational results

S3.4. Modulation depth analysis

S1. EXPERIMENTAL METHODS

S1.1. Synthesis

The syntheses of molecules **[1]** and **[2]** were previously reported by Oancea *et al.*[1] and Di Valentin *et al.*,[2] respectively.

S1.2. Sample preparation

All solvents were purchased from Sigma Aldrich. Solutions for ESR were prepared at a molecular concentration of 40 μM in d_6-ethanol for **[1]** and in a mixture of 98% d_4-methanol and 2% D_2O for **[2]**, and were loaded in 4 mm diameter quartz tubes to a sample height of 5 mm optimized to the size of the optical window of the cryostat. The solvents were chosen to improve solubility and favor helicity of the peptide backbones. Samples were degassed by several freeze-pump-thaw cycles and frozen in liquid nitrogen prior to insertion into the spectrometer.

S1.3. ESR spectroscopy

ESR experiments were performed at X-band (microwave frequency = 9.7 GHz) and a temperature of 20 K in a pulsed spectrometer (ElexSys E680, Bruker) using a dielectric resonator (EN 4118X-MD5, Bruker) and an Oxford Instruments cryostat. Laser 1 (OPOlette, Lambda Photometrics) was directed into the resonator through the optical window of the cryostat, while laser 2 (VersaScan OPO, GWU) was delivered via a 1 mm x 6 m optical fiber (FT1000-EMT, Thorlabs) directly inserted inside the sample tube. Both lasers were set at a wavelength of 512 nm, corresponding to the most intense maximum of the porphyrin Q-band region determined by UV-Vis. For the pulse experiments, a delay pulse generator was used to externally trigger both lasers and the spectrometer

at a repetition rate of 20 Hz, and the microwave pulses were moved forward in time with respect to the fixed laser flashes. All spectra were acquired with one shot per point.

Time-resolved ESR (TRESR) was carried out in a critically coupled resonator using Laser 1 only, without field modulation or phase sensitive detection. A microwave attenuation of 33 dB and a time base of 4 ns were used. The signal was averaged between 1.4 and 2.4 μs after the laser flash (256 time points), around the intensity maximum of the time trace. Triplet state parameters were extracted via simulation of the spectrum using the Matlab® *EasySpin* routine (*pepper* function).[3]

Electron spin-echo (ESE) experiments (i.e. field sweeps, T_2 and DAF experiments) were performed in an over-coupled resonator using a standard Hahn echo sequence preceded by a laser flash (laser 1 – DAF – π/2 – τ – π – τ – echo), with a length of 16 ns for the π/2 pulse. Field swept electron spin-echo spectra were recorded using a DAF of 1066 ns, a τ value of 320 ns, a laser energy of 2 mJ per flash and 3 scans. For transverse relaxation (T_2) experiments the τ value was incremented in steps of 16 ns and 30 scans were averaged. Delay after flash (DAF) experiments were carried out using a τ value of 200 ns, a laser energy of 3 mJ per flash and a total of 2 scans. The DAF was incremented in steps of 2000 ns starting with a value of 1566 ns. For both T_2 and DAF experiments the external magnetic field was set to the maximum of the field swept ESE spectrum (Y^- canonical orientation of the ZFS tensor, c.a. 335.1 mT).

LITTER traces were acquired using the following pulse sequence: laser 1 – DAF – π/2 – τ – π – τ' – laser 2 – τ'' – echo, with a length of 16 ns for the π/2 pulse. In all cases a τ = τ' + τ'' = 2004 ns was used and the DAF was incremented from an initial value of 1958 ns in steps of 8 ns to reach a maximum increment of +2800 ns. A longer trace with a +5600 ns increment was recorded in order to determine the zero time of the experiment. The average energies per laser flash were set

to 2 mJ (laser 1) and 2.5 mJ (laser 2), and the delay between flashes was t_{pp} = 6.82 μs. Measurements were carried out at three different values of the external magnetic field, corresponding to the Y^- and Z^- canonical orientations of the ZFS tensor and a position in between X^- and Y^- (c.a. 335.1, 386.0 and 333.4 mT, respectively). The total accumulation times for these measurements were 20, 93 and 20 h, respectively. The accumulation time for the experiments in figures S2 and S3 was 2h per trace. Raw LITTER traces were phase- and background-corrected to obtain the form factors, and those were analyzed via Fourier Transform and Tikhonov Regularization using the Matlab® *DeerAnalysis2018* routine to obtain the corresponding distance distributions.[4] The zero time used for the analysis (854 ns) was obtained from the full LITTER trace in Fig. 1 (b).

S2. COMPUTATIONAL METHODS

S2.1. Density Functional Theory calculations

Initial geometries for peptides **[1]** and **[2]** were built based on previously reported similar molecules,[2] using UCSF Chimera.[5] Geometry optimizations and spin density calculations were performed *in vacuo* using Gaussian® 09 (revision A.02).[6] Ground state geometry optimizations of peptides **[1]** and **[2]** were carried out in the singlet state, using the PBE1PBE functional and the 6-31g(d) basis set. Triplet state geometry optimizations of each of the TPP moieties from peptide **[1]** were subsequently performed using the functional B3LYP and the basis set SV(P). Electronic spin densities were obtained by single-point calculations on the TPP moieties previously optimized in the triplet state, using B3LYP and the basis set EPR-II.[7] Zero-Field Splitting (ZFS) tensor orientations for the two TPP moieties in peptide **[1]** in the triplet state were calculated using Orca

(release version 4.2.0),[8] with the functional B3LYP and the basis set EPR-II. The spin-spin contribution to the ZFS was calculated using computed UNO (spin-unrestricted natural orbital) determinants.[9]

S2.2. Orientation dependent simulations

Simulations of the LITTER data recorded on the Y^- and Z^- field positions were performed using a modified version of the algorithm reported by Lovett *et al.*,[10] to take into account the zero-field splitting and triplet populations. The spin system parameters were accounted for using *EasySpin* functions to simulate the spectra and resonant fields.[3] The input orientations were derived from the optimized structures of **[1]** *bent* and **[1]** *extended*. The position of the pump center with respect to the detection center was defined in terms of the polar angles (φ and θ) and a separation r, and the relative orientation of the zero-field splitting frames of the two centers was defined by the Euler angles (α,β,γ). Geometries in the range $\Delta\varphi = \pm15°$, $\Delta\theta = \pm15°$, $\Delta r = \pm0.5$ nm, $\Delta\alpha = \pm15°$, $\Delta\beta = \pm15°$ and $\Delta\gamma = \pm15°$ were sampled around the optimized structures of **[1]** *bent* and **[1]** *extended* to form two libraries of conformers. LITTER traces corresponding to the experimental parameters used for the detection on the Y^- and Z^- field positions were calculated for each orientation. These used a detection π pulse length of 32 ns, a detection frequency of 9.67 GHz and field positions of 335.1 mT and 386.0 mT respectively. The delocalization of the spin density and the orientation of the zero-field splitting tensors were taken from the DFT calculations. The parameters for the spin system used were found by fitting the TRESR spectrum of **[1]** (see Fig. S2). Orientation selection effects due to magnetophotselection of the porphyrin by the pump laser (laser 2) were not included in the simulations as this laser pulse was delivered via a fiber optic and it was assumed that all polarization was lost within this setup.

The libraries of simulated LITTER traces for **[1]** *bent* and **[1]** *extended* were fitted both independently and simultaneously to the experimental datasets using an algorithm similar to that described by Marko *et al.*[11] The results of these fitting procedures are shown in Figure 4 (main text) and Figures S10 and S11 (SI).

S3. RESULTS

S3.1. Spectroscopic characterisation of peptide [1]

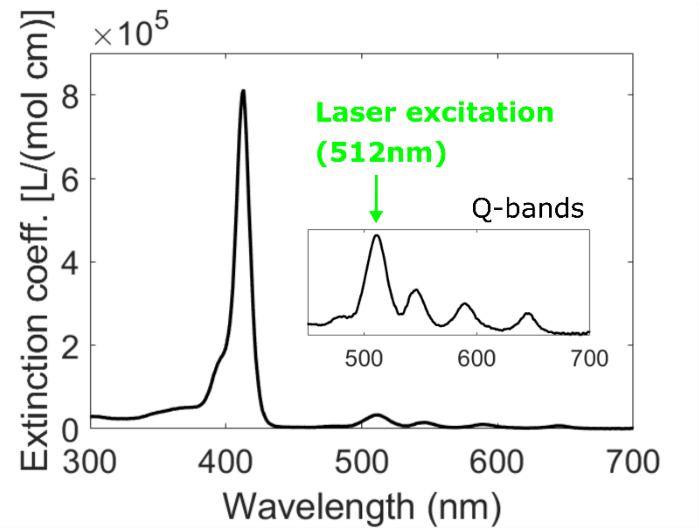

Figure S1. UV-Vis absorption spectrum of 14 μM **[1]** in a mixture of 98% d_4-methanol and 2% D_2O at room temperature. (inset) Zoom in the porphyrin Q-bands, indicating the laser excitation wavelength for the LITTER experiment, corresponding to the most intense maximum of the Q-band region. Spectrum measured using a Cary60 UV-Vis spectrometer (Agilent) and a 2.00 mm quartz cell.

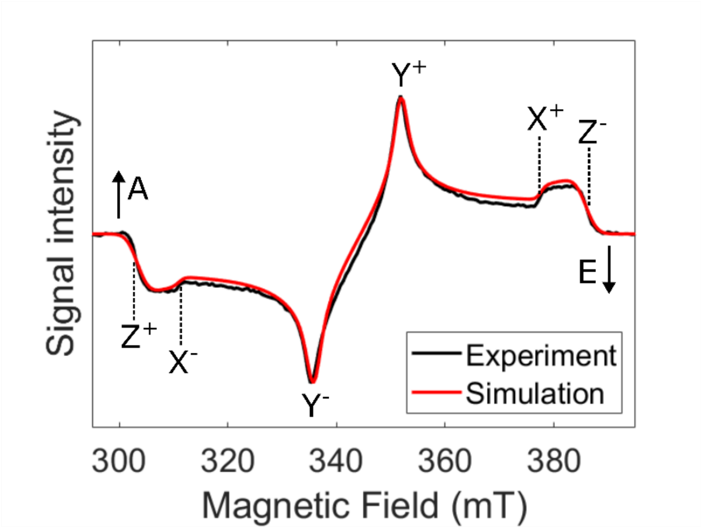

Figure S2. TRESR spectrum of **[1]** measured after laser flash (black) and simulation (red) using the following parameters: D = 41.4 mT, E = -8.4 mT, D-strain = 3.8 mT, E-strain = 0.0 mT, g = 2.006, linewidth = 0.7 mT and triplet state sublevel populations p_x = 0.32, p_y = 0.46 and p_z = 0.23. The turning points, corresponding to the canonical orientations of the anisotropic ZFS tensor, are labelled (Z^+, X^-, Y^-, Y^+, X^+ and Z^-, from left to right) and the absorptive (A) and emissive (E) parts of the spectrum are indicated.

The transverse spin relaxation time (T_2) of the photoexcited triplet state in **[1]** was similar to the τ value used in the experiment (Fig. S3 (a)), and both longitudinal spin relaxation time and triplet lifetime were much longer than the LITTER pulse sequence (Fig. S3 (b)), therefore only contributing to the background.

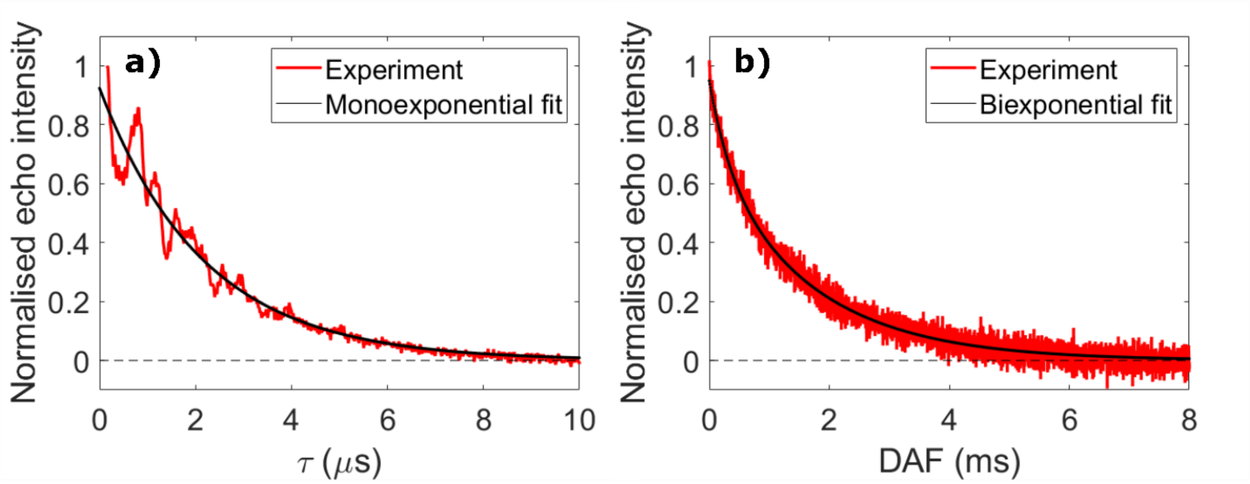

Figure S3. Relaxation experiments with molecule **[1]**. (a) Transverse relaxation experiment (red) and corresponding monoexponential fit (black) with a T_2 value of (2.15 ± 0.05) µs. (b) Delay after flash experiment (red) and corresponding biexponential fit (black) with lifetimes of (0.34 ± 0.04) ms and (1.68 ± 0.03) ms for the two exponentials.

S3.2. Optimisation of experimental conditions for LITTER

The effects of laser flash energy in signal intensity and modulation depth were studied (Fig. S4). Higher laser 1 energies improved signal intensity (Fig. S4 (a)), in consistence with the formation of more triplet spins before the Hahn echo sequence, but the signal was nearly saturated at 2 mJ. However, they did not cause any effective change in modulation depth (Fig. S4 (b)) as expected, therefore rendering better modulation-to-noise ratios (MNR) at higher energies. Higher laser 2 energies clearly improved modulation depth (Fig. S4 (d)), as expected from more triplet spins being formed during the Hahn echo sequence, but had a negative effect in signal intensity (Fig. S4 (c)). Two different factors seem to be contributing to the observed decrease in signal intensity at high laser 2 energies. First, the shorter T_2 due to the formation of more second triplets by laser 2

on molecules where a first triplet had already been formed by laser 1 (Fig. S5). Second, the incomplete decay of the photoexcited triplets back to the ground state due to the TPP triplet lifetime at cryogenic temperatures being comparable to the short repetition time (50 ms) used in the LITTER experiment, which becomes more important when larger numbers of triplet states are formed by larger laser energies. That would also be consistent with the nonlinearity in the laser 1 energy dependence of the signal intensity (Fig. S4 (a)). The sample was aerated in an attempt to increase signal intensity by reducing triplet lifetime. The signal was indeed slightly increased for very short τ values, but the improvement was lost for the $\tau \sim 2$ μs of the LITTER experiment, due to the reduction of T_2 caused by the presence of paramagnetic oxygen.

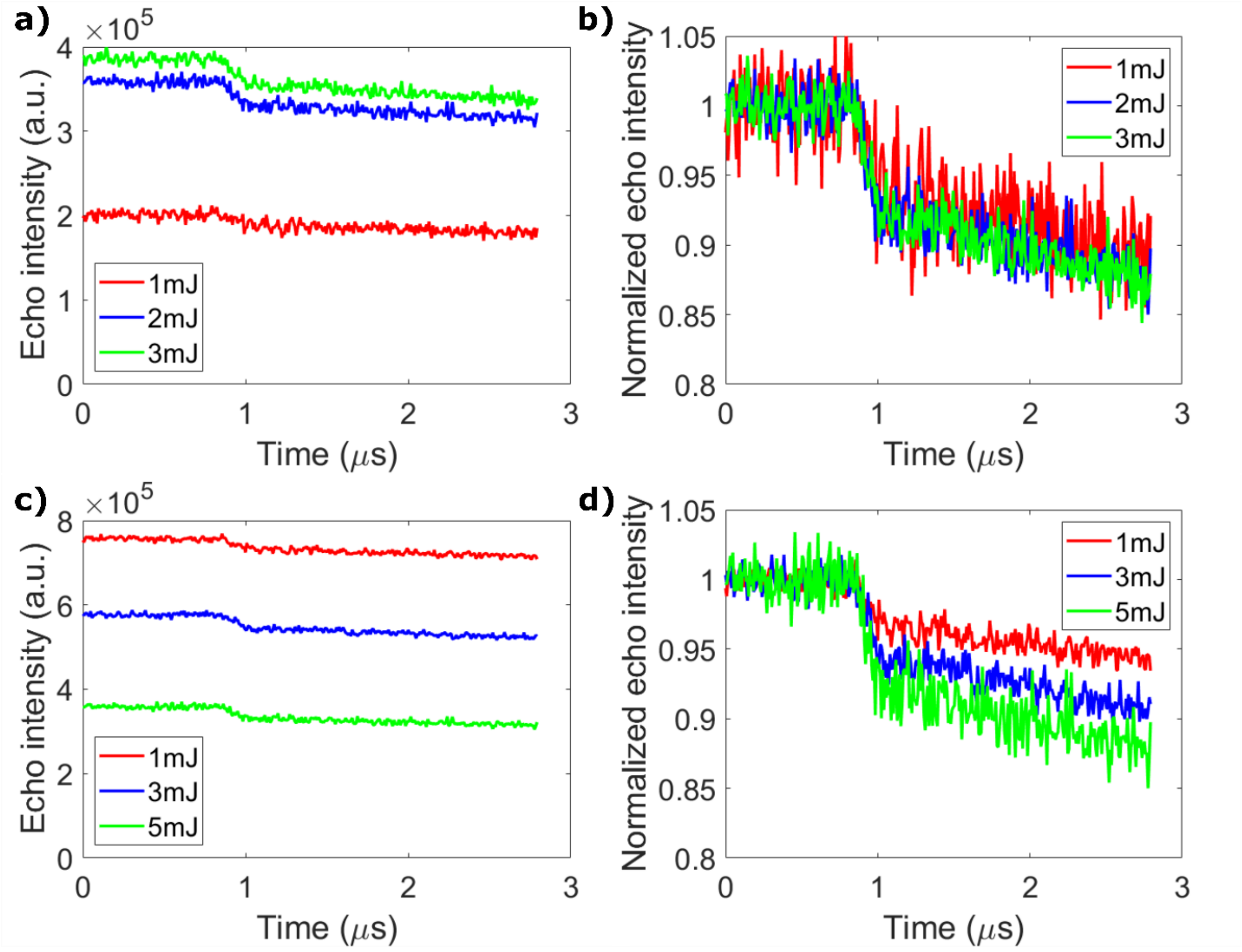

Figure S4. Laser energy studies. (a, b) LITTER traces measured with a Laser 2 energy of 5 mJ per flash and Laser 1 energies of 1 mJ (red), 2 mJ (blue) and 3 mJ (green) per flash. (c, d) LITTER traces measured with a Laser 1 energy of 2 mJ per flash and Laser 2 energies of 1 mJ (red), 3 mJ (blue) and 5 mJ (green) per flash. The data in (b) and (d) has been normalized to the start of the traces.

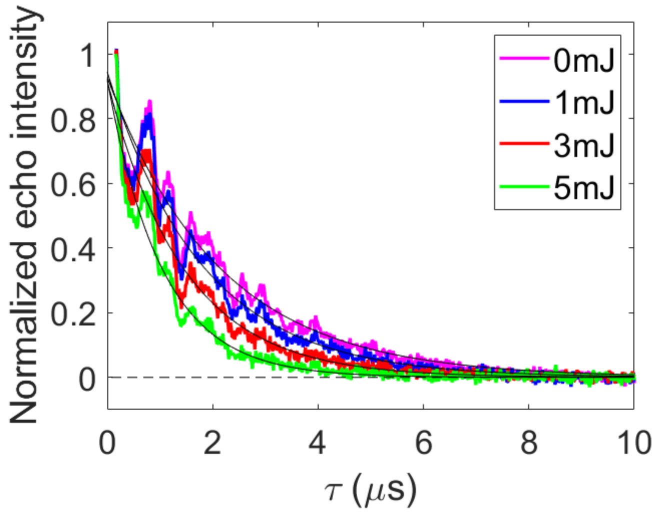

Figure S5. T_2 experiments at different laser 2 flash energies: 0 mJ (magenta), 1 mJ (blue), 3 mJ (red) and 5 mJ (green), with the corresponding monoexponential fits. The energy of laser 1 was fixed to 3 mJ per flash. Data has been normalized to the start of the traces.

The importance of a good overlap volume between the two laser beams for modulation depth was studied by changing the sample position and concentration (Fig. S6). In the optimized setup, the ~ 5 mm height sample is centered in the resonator and laser 1 is focused in a ~ 1 mm spot on the center of the sample. The sample above the volume excited by laser 1 absorbs light from laser 2 but does not contribute to the signal, reducing the number of laser 2 photons that reach the overlap volume (therefore it will be called "dead volume"). By pushing the sample tube further down into the resonator, the dead volume is reduced, leading to an increase in the number of laser 2 photons

that reach the overlap volume and a corresponding increase in modulation depth (Fig. S6 (b)), similar to effect of increasing laser 2 energy (Fig. S4 (d)). The decrease in signal intensity could be explained by a fraction of the laser 1 photons missing the sample, above the top.

Doubling the concentration of **[1]** from 40 μM to 80 μM in the optimized setup also doubled signal intensity because of twice as many molecules being photoexcited by laser 1 (Fig. S6 (c)). However, this also intensified the attenuation of laser 2 by the dead volume, leading to a significant decrease in modulation depth.

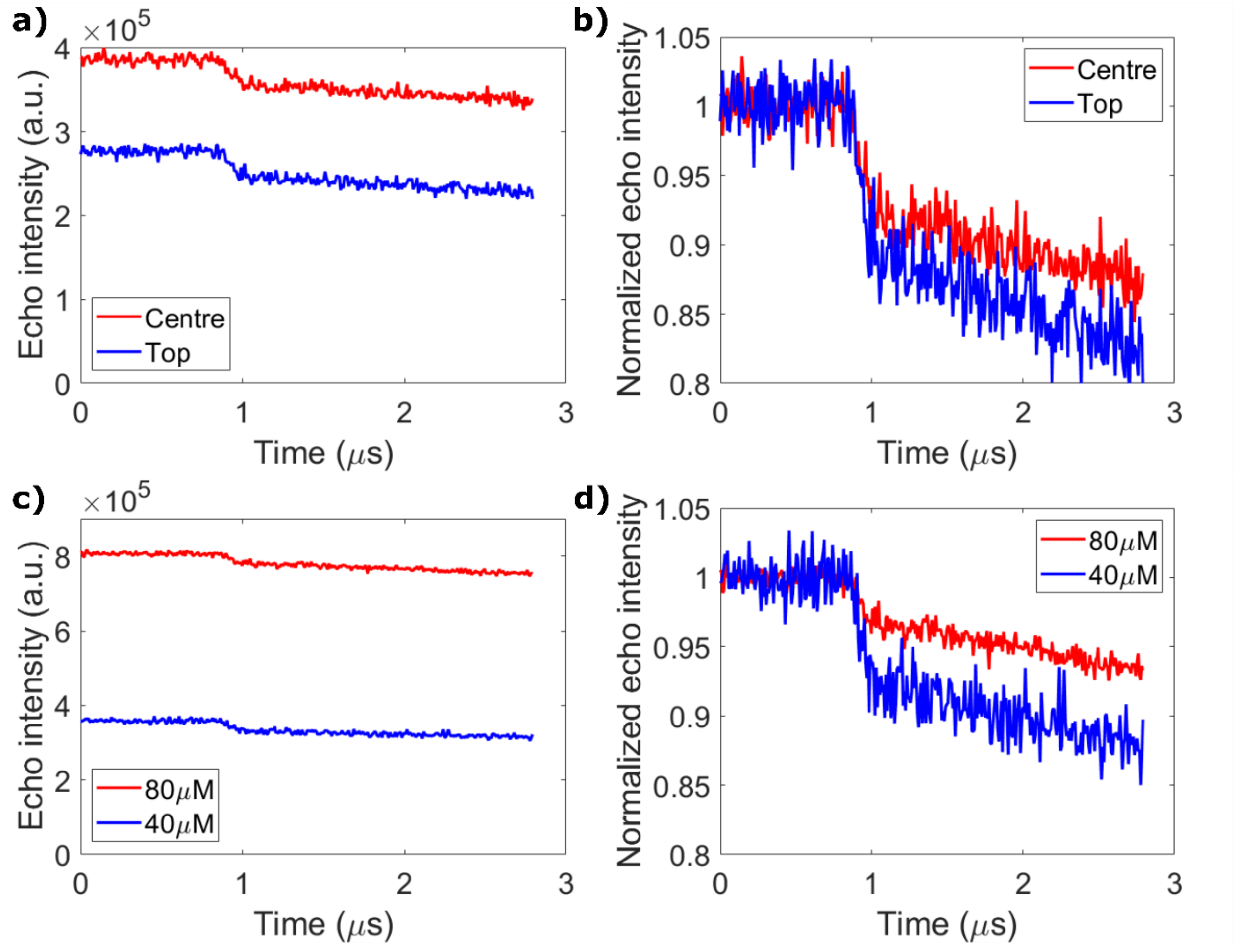

Figure S6. Sample position and concentration studies. (a, b) LITTER traces measured with the sample at two different positions, with Laser 1 hitting the center (red) and the top (blue) of the

sample. Laser energies per flash were 3 mJ (Laser 1) and 5 mJ (Laser 2). (c, d) LITTER traces for two different concentrations of molecule **[1]**: 80 μM (red) and 40 μM (blue). Laser energies per flash were 2 mJ (Laser 1) and 5 mJ (Laser 2). The data in (b) and (d) has been normalized to the start of the traces.

S3.3. Computational results

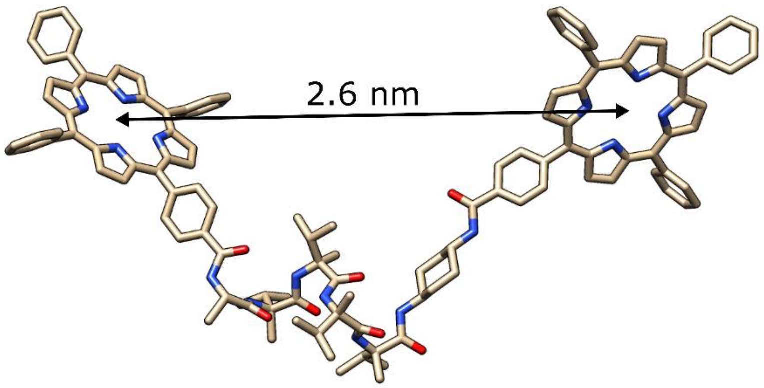

[1] *bent*

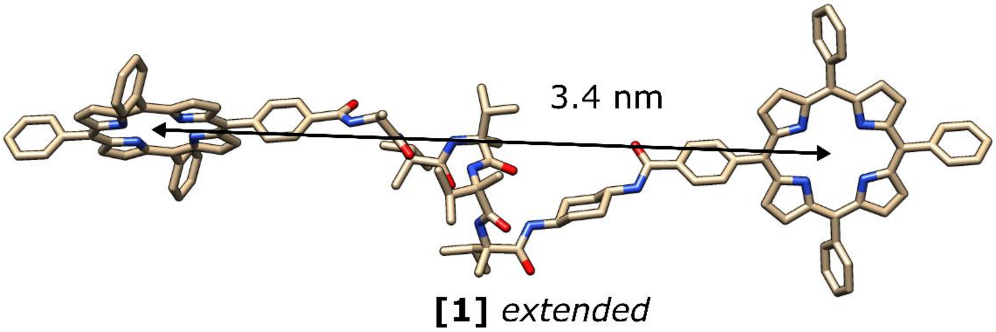

[1] *extended*

Figure S7. Ground state optimized geometries of the two local energy minima of peptide **[1]**, calculated using Gaussian 09 (PBE1PBE, 6-31g(d)). (top) **[1]** *bent* conformer (most stable), and

(bottom) **[1]** *extended* conformer, with center-to-center inter-porphyrin distances of 2.6 nm and 3.4 nm, respectively. Hydrogen atoms have been omitted in the figure for clarity. The calculated energy difference between the two geometries is 0.84 kJ/mol.

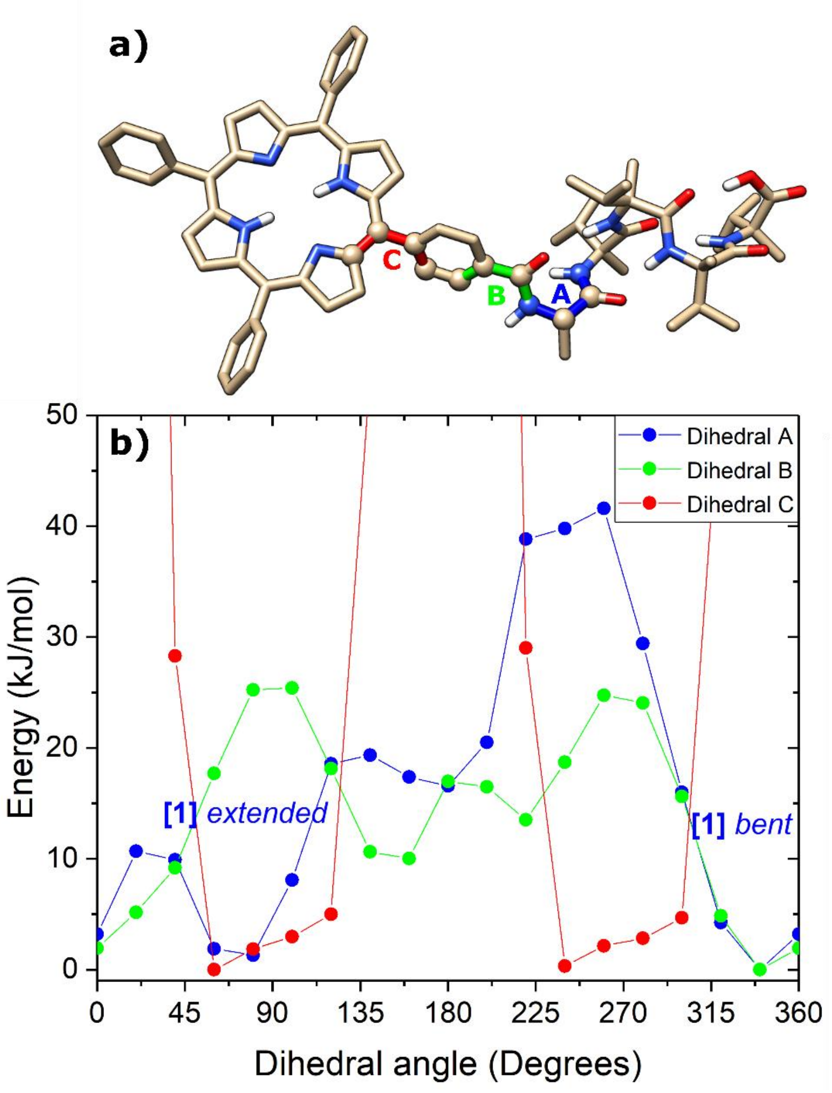

Figure S8. Energetics of bond rotation. (a) Structure of **[1]** *bent* conformer, where the second porphyrin has been omitted for clarity. The bonds defining the three dihedral angles studied are indicated as “A” (blue, rotation around the C^{α}-C^{amide} bond of the Ala, corresponding to the conversion between the *bent* and *extended* conformers), “B” (green, rotation around the C^{phenyl}-

C^{amide} bond) and “C” (red, rotation around the $C^{porphyrin}$-C^{phenyl} bond). (b) Energy profiles of the rotation around the abovementioned bonds, calculated using Gaussian 09 (PBE1PBE, 6-31g(d)). The two energy minima in the profile for dihedral A (blue) corresponding to the **[1]** *bent* and **[1]** *extended* conformers are indicated, with an energy barrier of interconversion of 11 kJ/mol. The absolute minimum of each energy profile has been assigned an energy of 0 kJ/mol.

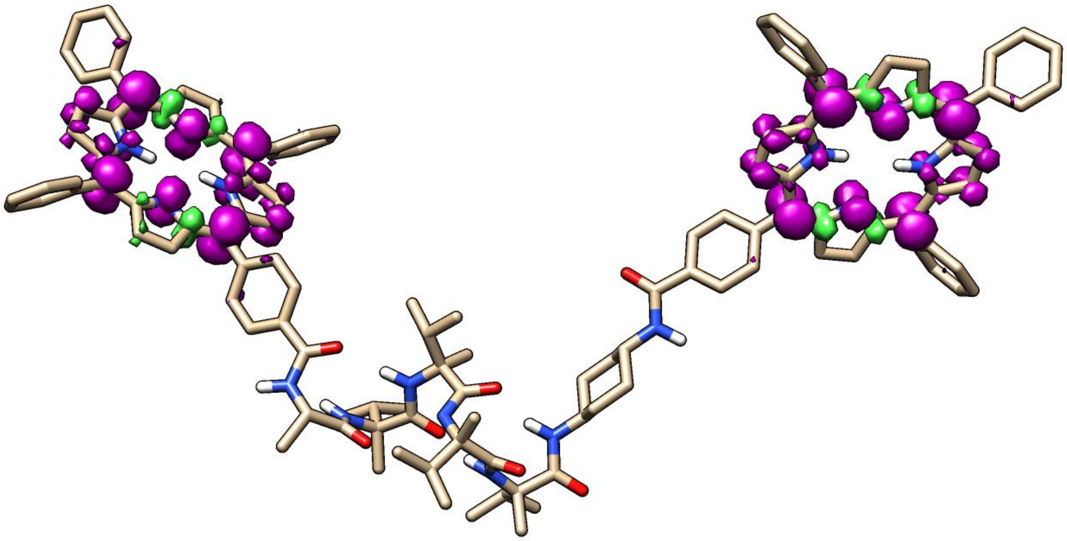

Figure S9. Electronic spin density in the DFT-optimized **[1]** *bent* conformer, where both TPP moieties have been photoexcited to the first triplet state, calculated using Gaussian 09 (B3LYP, EPR-II).

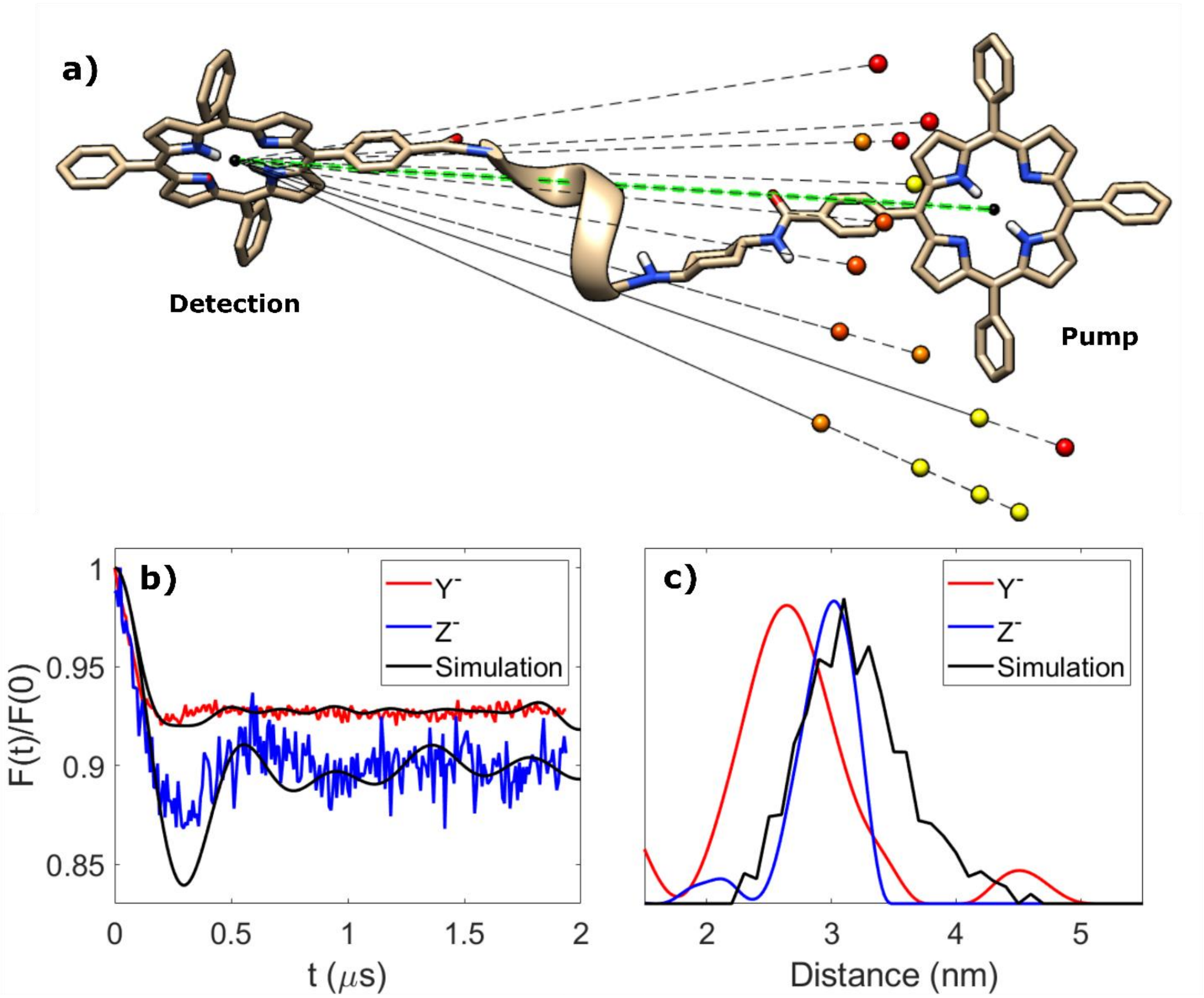

Figure S10. Orientation dependent LITTER simulation for **[1]** *extended* conformer. (a) DFT-optimized geometry with the different positions of the pump spin center contributing to the simulated form factor shown as spheres representing the centers of the corresponding porphyrins. The relative weight of each pump position in the simulated form factor is represented by the color of the sphere (yellow = 1, orange = 2, dark orange = 3 and red = 4). The dipolar vector between the centers of the two porphyrins of the DFT-optimized geometry is highlighted in green. Hydrogen atoms have been omitted in the figure for clarity. (b) Form factors obtained experimentally (red and blue) and from orientational dependent simulations (black). (d) Spin-spin

distance distributions obtained using *DeerAnalysis* (red and blue) and from orientational dependent simulations (black).

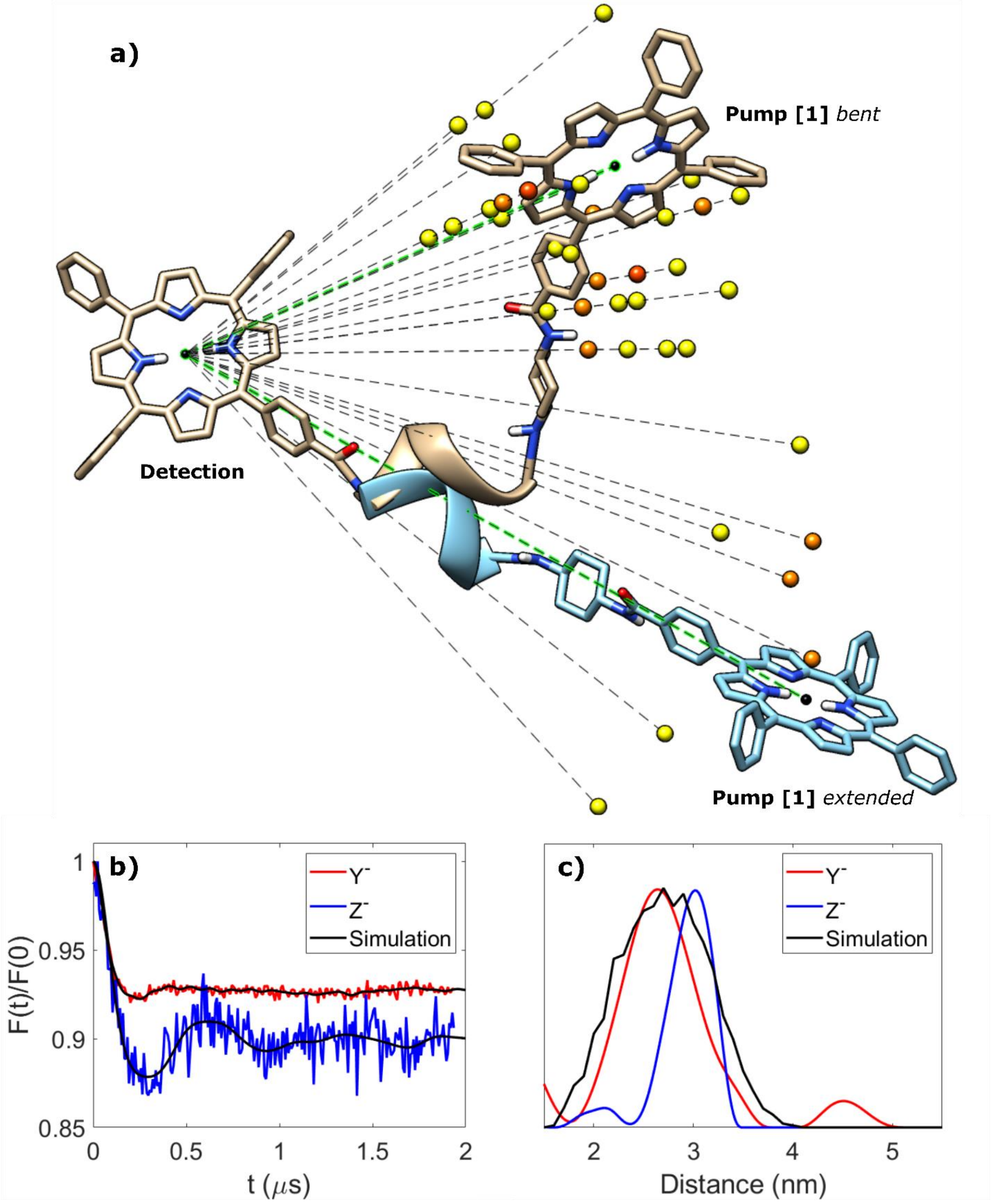

Figure S11. Orientation dependent LITTER simulation considering both **[1]** *bent* and **[1]** *extended* conformers simultaneously. (a) DFT-optimized geometries of **[1]** *bent* (brown) and **[1]** *extended* (blue), with the different positions of the pump spin center contributing to the simulated form factor shown as spheres representing the centers of the corresponding porphyrins. The relative weight of each pump position in the simulated form factor is represented by the color of the sphere (yellow = 1, orange = 2 and dark orange = 3). The dipolar vector between the centers of the two porphyrins of the DFT-optimized geometries is highlighted in green. Hydrogen atoms have been omitted in the figure for clarity. (b) Form factors obtained experimentally (red and blue) and from orientational dependent simulations (black). (d) Spin-spin distance distributions obtained using *DeerAnalysis* (red and blue) and from orientational dependent simulations (black).

S3.4. Modulation depth analysis

Assuming that all molecules have two porphyrin moieties that can be excited, the modulation depth observed will be a function of the porphyrin excitation efficiencies by laser 1 (e_1) and laser 2 (e_2), and the triplet state quantum yield (φ_T) of the porphyrin. This can be determined by calculating the proportion of molecules where both porphyrins are excited to the triplet state ($p_{\text{T-T\_laser1}}$), the proportion of molecules where only one porphyrin forms a triplet state ($p_{\text{S-T\_laser1}}$) and the proportion where neither porphyrin forms a triplet ($p_{\text{S-S\_laser1}}$) by laser 1. These are calculated as follows:

$$p_{\text{T-T\_laser1}} = (e_1 \phi_{\text{T}})^2$$

$$p_{\text{S-T\_laser1}} = 2e_1\phi_{\text{T}} \cdot (1 - e_1\phi_{\text{T}})$$

$$p_{S\text{-S\_laser1}} = (1 - e_1\phi_{\text{T}})^2$$

Molecules in $p_{\text{T-T\_laser1}}$ contribute to the observed echo intensity but cannot contribute to the modulation depth, while molecules in $p_{\text{S-T\_laser1}}$ contribute to the observed echo intensity and may contribute to the modulation depth if the singlet porphyrin is excited by the second laser. Molecules that were not excited by the first laser, $p_{\text{S-S\_laser1}}$, will not contribute to the echo intensity or the modulation depth. After the second laser flash, the overall proportion of molecules remaining with only one moiety excited is $p_{\text{S-T\_laser2}}$. These molecules will contribute to the echo intensity but will not contribute to the modulation depth. Molecules where the first laser excites one porphyrin to the triplet and the second laser excites the other porphyrin to the triplet, $p_{\text{T-T\_laser2}}$, will contribute both to the observed echo intensity and to the modulation depth. The populations of each of these states is given by

$$p_{\text{S-T\_laser2}} = p_{\text{S-T\_laser1}} \cdot (1 - e_2\phi_{\text{T}})$$

$$p_{\text{T-T\_laser2}} = p_{\text{S-T\_laser1}} \cdot e_2\phi_{\text{T}}$$

where

$$p_{\text{S-T\_laser1}} = p_{\text{S-T\_laser2}} + p_{\text{T-T\_laser2}}$$

The echo intensity at the zero time of the experiment (S_0) is proportional to the fraction of molecules where a triplet state has been formed by the first laser, and the modulated part of the signal (S_M) is proportional to $p_{\text{T-T\_laser2}}$, such that

$$S_0 = p_{\text{S-T\_laser1}} + 2p_{\text{T-T\_laser1}}$$

$$S_{\text{M}} = p_{\text{T-T\_laser2}}$$

The modulation depth, Δ, is thus given by

$$\Delta = \frac{S_{\mathrm{M}}}{S_0} = e_2\phi_{\mathrm{T}} \cdot (1 - e_1\phi_{\mathrm{T}})$$

Porphyrin excitation efficiencies e_1 and e_2 can be estimated from the absorbance of the sample at the wavelength of irradiation, the laser pulse energies used and the geometry of the experiment. In our case we have estimated that the pulse energies for both laser 1 and laser 2 are high enough to optically saturate the overlap volume, making $e_1 = e_2 = 1$. This is consistent with the marginal change in echo intensity observed when increasing the energy of laser 1 from 2 mJ to 3 mJ (Fig. S4 (a)). Using the reported value of $\varphi_T = 0.8$ for free-base TPP,[12] we predict a modulation depth Δ = 0.16, which is slightly larger than our measured values. This difference likely originates from experimental factors such as the background correction used in the data analysis, as the background decay used impacts the measured modulation depth, the fact that porphyrin excitation efficiencies may not be exactly 1, or the fact that the second laser may not excite completely the portion of the sample excited by the first laser. Furthermore, the calculation above assumes that all molecules have two moieties that can be excited. This assumption may not be experimentally appropriate as sample bleaching can occur and was observed during our experiments as a decrease in both echo intensity and modulation depth over time.

REFERENCES

(1) Oancea, S.; Formaggio, F.; Campestrini, S.; Broxterman, Q. B.; Kaptein, B.; Toniolo, C. Distance Dependency of Exciton Coupled Circular Dichroism Using Turn and Helical

Peptide Spacers. *Biopolymers* **2003**, *72* (2), 105–115. https://doi.org/10.1002/bip.10315.

(2) Di Valentin, M.; Albertini, M.; Dal Farra, M. G.; Zurlo, E.; Orian, L.; Polimeno, A.; Gobbo, M.; Carbonera, D. Light-Induced Porphyrin-Based Spectroscopic Ruler for Nanometer Distance Measurements. *Chem. - A Eur. J.* **2016**, *22* (48), 17204–17214. https://doi.org/10.1002/chem.201603666.

(3) Stoll, S.; Schweiger, A. EasySpin, a Comprehensive Software Package for Spectral Simulation and Analysis in EPR. *J. Magn. Reson.* **2006**, *178* (1), 42–55. https://doi.org/10.1016/j.jmr.2005.08.013.

(4) Jeschke, G.; Chechik, V.; Ionita, P.; Godt, A.; Zimmermann, H.; Banham, J.; Timmel, C. R.; Hilger, D.; Jung, H. DeerAnalysis2006 - A Comprehensive Software Package for Analyzing Pulsed ELDOR Data. *Appl. Magn. Reson.* **2006**, *30* (3–4), 473–498. https://doi.org/10.1007/BF03166213.

(5) Pettersen, E. F.; Goddard, T. D.; Huang, C. C.; Couch, G. S.; Greenblatt, D. M.; Meng, E. C.; Ferrin, T. E. UCSF Chimera - A Visualization System for Exploratory Research and Analysis. *J. Comput. Chem.* **2004**, *25* (13), 1605–1612. https://doi.org/10.1002/jcc.20084.

(6) Frisch, M. J.; Trucks, G. W.; Schlegel, H. B.; Scuseria, G. E.; Robb, M. A.; Cheeseman, J. R.; Scalmani, G.; Barone, V.; Petersson, G. A.; Nakatsuji, H.; et al. Gaussian 09. Gaussian, Inc.: Wallingford CT 2009.

(7) Barone., V. Structure, Magnetic Properties and Reactivities of Open-Shell Species from Density Functional and Self-Consistent Hybrid Methods. In *Recent Advances in Density Functional Methods, Part I*; Chong, D. P., Ed.; World Scientific Publ. Co.: Singapore, 1996.

(8) Neese, F. The ORCA Program System. *Wiley Interdiscip. Rev. Comput. Mol. Sci.* **2012**, *2* (1), 73–78. https://doi.org/10.1002/wcms.81.

(9) Sinnecker, S.; Neese, F. Spin-Spin Contributions to the Zero-Field Splitting Tensor in Organic Triplets, Carbenes and Biradicals - A Density Functional and Ab Initio Study. *J. Phys. Chem. A* **2006**, *110* (44), 12267–12275. https://doi.org/10.1021/jp0643303.

(10) Lovett, J. E.; Bowen, A. M.; Timmel, C. R.; Jones, M. W.; Dilworth, J. R.; Caprotti, D.; Bell, S. G.; Wong, L. L.; Harmer, J. Structural Information from Orientationally Selective DEER Spectroscopy. *Phys. Chem. Chem. Phys.* **2009**, *11*, 6840–6848. https://doi.org/10.1039/b913085n.

(11) Marko, A.; Prisner, T. F. An Algorithm to Analyze PELDOR Data of Rigid Spin Label Pairs. *Phys. Chem. Chem. Phys.* **2013**, *15* (2), 619–627. https://doi.org/10.1039/c2cp42942j.

(12) Murov, S. L.; Carmichael, I.; Hug, G. L. *Handbook of Photochemistry*, Second.; Marcel Dekker, Inc.: New York, 1993.
